# Relationship between Pre-Procedural Serum Lipid Profile and Post-Procedural Myocardial Injury in Patients Undergoing Elective Percutaneous Coronary Intervention

**DOI:** 10.5812/cardiovascmed.11542

**Published:** 2013-10-28

**Authors:** Mohsen Maadani, Seifollah Abdi, Sepideh Parchami-Ghazaee, Keivan Alizadeh, Hosein Fathi, Reza Musavi

**Affiliations:** 1 Cardiovascular Intervention Research Center, Rajaie Cardiovascular Medical and Research Center, Iran University of Medical Sciences, Tehran, IR Iran

**Keywords:** Angioplasty, Lipids, Myocardial Reperfusion Injury

## Abstract

**Background:**

Along with technological progress in coronary intervention, periprocedural complications and adverse outcomes have markedly improved, yet perioperative myocardial injury is a frequent complication during percutaneous coronary intervention (PCI) and is strongly associated with post-procedural cardiovascular morbidity and mortality. Epidemiological researchers have defined lipid and lipoproteins abnormality as a risk factor for atherosclerotic cardiovascular diseases. Although several studies focus on identification the correlation between the changes of lipid profile levels and ischemic markers, there is a little information about the role of lipid profile disturbance as a predictor of periprocedural myocardial injuries.

**Objectives:**

This study aimed to observe the relationship between lipid profile levels and the post-procedural myocardial injury in patients undergoing elective PCI.

**Patients and Methods:**

This case-control study was conducted on 138 consecutive patients with a diagnosis of coronary artery disease who underwent PCI. Of a total 138, 35 patients had cardiac biomarker elevation, more than 3 × ULN, post-procedurally. The control group (n = 103), without cardiac enzyme rising after PCI were randomly chosen three times the number of patients with increased cardiac enzymes more than three times the ULN. Samples for serum lipid parameters [total cholesterol (TC), low-density lipoprotein cholesterol (LDL-C), high-density lipoprotein cholesterol (HDL-C), triglyceride (TG), and very low-density lipoprotein cholesterol (VLDL)] were collected after 12-14 fasting hours immediately pre-procedurally. The samples for CPK-MB were collected at 8, 16, and 24 hours post procedurally.

**Results:**

Although the mean level of TC, LDL-C and TG was higher in patients with CPK-MB more than 3×ULN post procedurally, differences were insignificant. Among different lipid parameters, only the mean level of VLDL showed a considerable association with myocardial injury. Although, this subject had a near significant (P = 0.05) enhancement in group I, the changes were in normal ranges. Lipid abnormality (except for the VLDL values) was insignificantly more frequent in group I.

**Conclusions:**

Although the mean level of non-HDL-C was in normal ranges, it showed a higher value in patients with a diagnosis of myocardial injury post procedurally. However, according to multivariate analysis, left ventricular ejection fraction and diabetes remained as predictors of post-procedural CPK-MB elevation.

## 1. Backgrounds

Routine percutaneous coronary intervention (PCI) appears to be a relatively safe catheterization procedure. Along with technological progress in coronary intervention, periprocedural complications and adverse outcomes have markedly improved, yet perioperative myocardial injury, manifested as myocardial stunning or infarction, is a frequent complication during PCI and is strongly associated with post-procedural cardiovascular morbidity and mortality (1). According to the World Health Organization one of the criteria for definition of myocardial infarction (MI) is cardiac biomarker elevation (2). It has been reported that post procedure creatinine-MB fraction (CPK-MB) elevation occurs approximately in 25% of patients and troponin I (TnI) level rising is observed at least in 50% of those undergoing PCI (3). Epidemiological researchers have defined lipid and lipoproteins abnormality as a risk factor for atherosclerotic cardiovascular disease (4). Moreover, several studies focus on identification the correlation between the changes of lipid profile levels and ischemic markers (5). A number of patient-related factors including diabetes mellitus, older age and chronic kidney disease are known to associate with periprocedural MI after PCI (2). However, there is a little information about the role of lipid profile disturbance as a predictor of periprocedural myocardial injuries. The results of the present study may propose association between dislipidemia and worsened prognosis following PCI.

## 2. Objectives

This study aimed to observe the relationship between lipid profile levels and the post-procedural myocardial injury in patients undergoing elective percutaneous coronary intervention.

## 3. Patients and Methods 

This case-control study was conducted on 138 consecutive patients with a diagnosis of coronary artery disease who underwent PCI between March 2012 and February 2013 at Rajaie Cardiovascular Medical, and Research Center, Iran University of Medical Sciences in Tehran. The inclusion criteria were a normal pre-procedural troponin I (TnI) level and creatinine-MB fraction (CPK-MB). Patients with a diagnosis of recent MI were excluded. The diagnosis of a pre-procedural MI was based on either the development of new pathological Q waves in at least two contiguous ECG leads or an elevation of CPK-MB > five times the upper limit of normal (ULN) or Tn > 0.06 µU/L. Of 138, 35 patients had cardiac biomarker elevation more than 3 × ULN post procedurally. The control group (n = 103), without cardiac enzyme rising after PCI were randomly chosen three times the number of patients with elevated cardiac enzymes. All the patients were treated according to the latest PCI guidelines, recommending Aspirin, Clopidogrel, and Atorvastatin at low to intermediate doses (6). According to medical treatment, patients who were treated with statins for at least one month and beta-blockers for two weeks before PCI were considered as statin or beta-blocker users. Samples for serum lipid parameters [total cholesterol (TC), low-density lipoprotein cholesterol (LDL-C), high-density lipoprotein cholesterol (HDL-C), triglyceride (TG) and very low-density lipoprotein cholesterol (VLDL)] were collected after 12-14 fasting hours immediately pre-procedurally and measured via the enzymatic colorimetric method with a biochemistry auto analyzer (Hitachi 917). The normal ranges for serum lipids were considered as follows: TC < 200 mg/dl; LDL-C < 130 mg/dl; HDL-C > 40 mg/dl for female, HDL-C > 35 mg/dl for male, and VLDLs < 38mg/dl (7). The samples for CPK-MB were collected at 8, 16, and 24 hours post-procedurally. The CPK-MB levels were measured via an enzymatic method (Biochemical auto analyzer). The ULN for CPK-MB was 25 IU/L. The standard PCI technology was utilized. All the patients provided written, informed consent to study participation, and the study protocol was approved by the institutional Review Board and the Ethics Committee of Tehran University of Medical Sciences. The statistical analyses were performed with SPSS software (SPSS 15 for Windows, SPSS Inc., Chicago, Illinois). The data were expressed as mean ± SD for the continuous and as percentages for the discrete variables. The independent samples t test or Mann Whitney U test was used to compare the continuous variables between the groups. The chi-square test was employed for the statistical analysis of the categorical variables, and the categorical variables were compared using the Fisher exact test, as appropriate. A logistic regression model was built including the dependent and independent variables with significance or near significance. Odds ratios and 95% confidence intervals (CI) were presented with two-tailed p values. P values < 0.05 were considered statistically significant.

## 4. Results

This study was conducted on 138 consecutive patients with a diagnosis of coronary artery disease who underwent PCI, 77 (55.8%) male and 61 (44.2%) female, with mean age of 60.05 ± 10.79 years and the total average of CPK-MB: 22.98 ± 10.74 IU/L before procedure and 29 ± 12.24 IU/L post procedurally. Demographic characteristics of patients in two groups are demonstrated in Table 1. The patients with a diagnosis of myocardial injury after procedure were significantly more likely to be diabetic ones. However, cigarette smoking prevalence was insignificantly lower in this group (Table 1). 

**Table 1.** Baseline Demographic Characteristics of Patients in Two Groups Undergoing PCI

Characteristics	CK-MB ≥ 3×ULN (n = 35)	Control Group (n = 103)	P value
**Age, y**	61.28 ± 10.9 ^a^	59.12 ± 9.9	0.283
**Gender, male**	24 (68.6)	79 (76.7)	0.340
**Diabetes**	12 (34.3)	15 (14.6)	0.011
**Hypertension**	16 (45.7)	42 (40.8)	0.604
**hyperlipidemia**	16 (45.7)	35 (34)	0.218
**Smoking**	7 (20)	39 (35.9)	0.081
**Family history of CAD^b^**	4 (11.4)	21 (20.4)	0.237

^a^ Values are expressed as mean ± SD or No. (%).

^b^ Abbreviation: CAD, coronary artery disease

According to medical treatment, there was not any reliable difference between frequency of statin and beta-blocker consumption in two groups. Data analysis in two groups depicted that the average of the left ventricular ejection fraction in the group with cardiac enzyme rising more than 3 × ULN was significantly lower than control group (45.28 ± 7.6 % in first group versus 49.10 ± 8.5% in control group; P = 0.005). Cardiac enzyme analysis revealed that the mean level of CPK-MB was 53.86 ± 30.86 IU/L in patients with enzyme elevation more than 3× ULN that was significantly more than whom without a diagnosis of myocardial injury after procedure (25.92 ± 68; P < 0.001). According to biochemistry analysis of serum samples the mean level of lipid profile parameters in 138 patients was as follows: TC=165.2 ± 56.3mg/dl, TG = 163.1 ± 95.7 mg/dl, LDL-C = 99.5 ± 47.4 mg/dl, HDL-C = 37.1 ± 9.05 mg/dl and VLDL = 28.7 ± 18.5 mg/dl. As demonstrated in Table 2 although the mean level of TC, LDL-C and TG was higher in patients with CPK-MB more than 3×ULN post procedurally, differences were insignificant. Among different lipid parameters, only the mean level of VLDL showed a considerable association with myocardial injury; although this subject was near significantly (P = 0.05) enhanced in this group, the changes were in normal ranges. 

**Table 2.** Serum Lipid Profile Levels in Two Groups Undergoing PCI

Lipid Parameters	CKMB≥3×ULN (n = 35)	Control Group (n = 103)	P value
**TC^a^** ** mg/dL**	174.45 ± 51.4 ^b^	162 ± 57.8	0.201
**LDL-C, mg/dL**	103.82 ± 35.8	98.11 ± 51.03	0.265
**HDL-C, mg/dL**	37.31 ± 9.6	37.03 ± 8.8	0.872
**TG mg/dL**	184.17 ± 13.5	155.98 ± 78.5	0.376
**VLDL mg/dL**	35.32 ± 24.8	26.48 ± 15.4	0.05

^a^ Abbreviations: HDL_C, high-density lipoprotein cholesterol; LDL_C, low-density lipoprotein cholesterol; TC, total cholesterol; TG, triglyceride; VLDL, very low-density lipoprotein cholesterol

^b^ Values are expressed as mean ± SD.

Table 3 reveals the prevalence of patients in various groups according to lipid profile parameters in different ranges. Lipid abnormality (except the VLDL values) was insignificantly more frequent in patients with elevated cardiac enzymes more than three times the ULN. A logistic regression model was applied to adjust for some covariates. 

**Table 3.** Prevalence of Patients in Two Groups Undergoing PCI According to Different Ranges of Lipid Profile Parameters

Lipid parameters, mg/dL	CK-MB ≥ 3×ULN (n = 35)	Control Group (n = 103)	P value
**TC ^a^(> 200 )**	10 (28.6) ^b^	18 (17.5)	0.167
**LDL-C (> 130) **	9 (25.7)	17 (16.5)	0.313
**HDL-C (< 35 M, < 40 F)**	20 (57.1)	55 (53.4)	0.703
**TG ( > 200 mg/dL)**	9 (25.7)	22 (21.4)	0.598
**VLDL (mg/dL > 38)**	12 (46.2)	14 (53.8)	0.007

^a^ Abbreviation: HDL_C, high-density lipoprotein cholesterol; LDL_C, low-density lipoprotein cholesterol; TC, total cholesterol; TG, triglyceride; VLDL, very low-density lipoprotein cholesterol

^b^ Values are expressed as No. (%)

The analysis for LDL-C identified that left ventricular ejection fraction and diabetes remained as predictors of post-procedural CPKMB elevation (Table 4). We mention that the results of the logistic regression for different characteristics of serum lipids were the same. 

**Table 4.** Multivariate logistic regression model for LDL-C

Covariates	β	P value	OR (CI 95%)
**LDL-C ** ^**a**^	-0.002	0.62	0.99 (0.98-1)
**Age**	-0.011	0.61	0.98 (0.94-1.03)
**Gender**	0.054	0.91	1.05 (0.41-2.71)
**LVEF**	-0.051	0.03	1.05 (1-1.10)
**Diabetes**	0.96	0.04	2.62 (1.02-6.71)
**Hyperlipidemia**	0.59	0.19	1.80 (0.74-4.36)
**Statin consumption**	-0.22	0.62	0.79 (0.32-1.95)

^a^ Abbreviations: LDL_C, Low-density lipoprotein cholesterol; LVEF, left ventricular ejection fraction

## 5. Discussion 

In our investigation we compared serum lipid profiles between two groups of patients who underwent PCI, with CPK-MB elevation more than 3× ULN, without a diagnosis of myocardial necrosis after procedure. It is evident that cardiac biomarkers have diagnostic and prognostic utility for myocardial injury (8). The type, extent and timing of biomarker release have been proposed in the Universal Definition of Myocardial Infarction (9). CPK-MB rising has been validated as a biomarker for definition of peri procedural MI and CPK-MB elevation ≥3× ULN is the most common predictor (3). However, numerous studies have demonstrated that even low levels of CPK-MB elevation have been associated with discrete micro infarcted areas (3, 10). Some studies claim that CPK-MB elevation in parallel to other risk factors like older age and presence of other medical problems can adversely affect short- or long-term prognosis (11). Dislipidemia that is observed in myocardial infarction patients and characterized by elevated TC, LDL-C and lowered HDL-C, is a recognized risk factor that synergistically acts with other non-lipid risk factor (12). In the present study we found that the mean level of non-HDL-C was insignificantly higher in patients with increased cardiac biomarkers, more than three times the ULN than the other group, although the levels were within normal lipid profile. Lipid abnormality is one of the risk factors resulting in the plaque formation due to excess cholesterol. The plaques that are deposited on the coronary wall vessels reduce blood flow causing ischemia and consequently damage myocardial cells. After myocyte damage, cellular proteins including myoglobin, lactate dehydrogenase, cardiac CPK and troponin (T and I) are released into the circulation (2, 5). Kumar and Sivakanesan have reported that the mean level of TC, LDL-C and TG were markedly greater in MI patients than non- MI subjects; On the contrary, the mean serum HDL-C level was significantly lower in patients with MI (13). However, in our study two groups were on the same average level according to HDL-C. Plasma triglyceride that is mainly carried by VLDLs is a significant predictor of cardiac events, (14). Unexpectedly the cholesterol enriched of VLDL particles is inversely related to risk of coronary events. Although this may be appear to be an atherogenic character of VLDL, rapid uptake of cholesterol-rich VLDL by LDL receptors in liver and its clearance is a protective manner against their entry into the arterial intima (15). In the present study although when two groups were considered related to VLDL ranging, the prevalence of patients with abnormal values in control group were markedly greater than the other group which means the mean level of VLDLs was higher in patients with post procedure myocardial injury. We note that assaying the VLDL type may be an important variable to determine independent predictors of coronary events. Although the mean level of non-HDL-C was in normal ranges, was higher in patients with a diagnosis of myocardial injury post procedurally. However, according to multivariate analysis, left ventricular ejection fraction and diabetes remained as predictors of post-procedural CPKMB elevation. Because peri-procedural enzyme rising may be related to many clinical, anatomical and procedural confounding factors including characteristics of culprit lesion and complications during PCI, it will be important to introduce these data in different groups and present bias-adjusted results for future studies.
